# A combination of plant-based compounds and extracts acts nematicidal and induces resistance against *Meloidogyne incognita* in tomato

**DOI:** 10.3389/fpls.2024.1411825

**Published:** 2024-07-04

**Authors:** Eva Degroote, Chloë Schoorens, Stefaan Pockelé, Boris Stojilković, Kristof Demeestere, Sven Mangelinckx, Tina Kyndt

**Affiliations:** ^1^ Department of Biotechnology, Faculty of Bioscience Engineering, Ghent University, Ghent, Belgium; ^2^ Synthesis, Bioresources and Bioorganic Chemistry Research Group, Department of Green Chemistry and Technology, Faculty of Bioscience Engineering, Ghent University, Research and Development, Ghent, Belgium; ^3^ Research and Development, Lima Europe NV, Rumst, Belgium; ^4^ Research Group Environmental Organic Chemistry and Technology (EnVOC), Department of Green Chemistry and Technology, Faculty of Bioscience Engineering, Ghent University, Ghent, Belgium

**Keywords:** nematicide, salvia extract, salicylic acid, ascorbic acid, geraniol, garlic extract, *Meloidogyne incognita*, *Solanum lycopersicum*

## Abstract

Considering the stricter European regulations for chemical pesticides (e.g. abolishment of the use of chemical soil fumigation products, such as methyl bromide), the need for more sustainable plant protection products is strongly increasing. In this research, Product X, an innovative mixture of bio-nematicidal compounds was developed and evaluated for efficacy. Product X showed a direct nematicidal effect against the root-knot nematode *Meloidogyne incognita*. In pot trials with tomato plants infected with *M. incognita*, Product X treatment lead to a significant reduction in nematode-induced gall formation. mRNA-sequencing indicated alterations in phytohormone levels and ROS-metabolism in tomato roots upon treatment with Product X, which was subsequently biochemically validated. Increased levels of abscisic acid and peroxidase activity seem to be the main factors in the response of tomato plants to Product X. Long-term administration of Product X did not yield negative effects on tomato growth or yield. In conclusion, Product X provides a new interesting mix of bio-active compounds in the combat against root-knot nematodes.

## 1 Introduction

Climate change is altering the world at a quick pace, due to global changes such as increased temperatures some plant pathogens are becoming more abundant and more prevalent in different crops and areas around the world (Dutta and Phani, 2023). Additionally, sustainability gains importance throughout society. The European Green Deal is an international initiative to improve sustainability and resource-efficiency in the European Union (European Commission, 2019). Within the European Green Deal, the ‘Farm to Fork’ strategy aims to make food production systems fair, healthy, and environmentally-friendly (European Commission, 2020). Additionally, ecologically harmful chemical pesticides are progressively banned or put on a substitution list by the European Food Safety Authority (EFSA). Products on the substitution list need to be replaced when adequate alternatives become available. This results in an urgent need for sustainable and innovative pesticides and plant protection products (PPPs).

PPPs remain a valuable part of Integrated Pest Management (IPM) programs to keep populations of e.g. damaging insects, bacteria, fungi or nematodes under economic injury levels. Although often not recognized as the main culprit due to their generic symptoms and subterranean location, nematodes or roundworms cause an annual worldwide average crop loss of 12.3% (Talavera-Rubia et al., 2022). Among the thousands of plant-parasitic nematode species present in agricultural ecosystems, root-knot nematodes (*Meloidogyne* spp.) are most damaging to crop plants. They penetrate the plant root as second-stage juveniles (J2) (Abad et al., 2008) and induce the formation of a feeding site consisting of giant cells inside the vascular tissue. Via the feeding sites, nematodes withdraw nutrients from the host and interfere with water transport (Abad et al., 2008). Root-knot nematode infection results in the formation of so-called root-knots or galls, which are the most visible symptom of the disease (Abad et al., 2008). Plant-parasitic nematodes are often managed with chemical products (nematicides and fumigants), some of which are harmful to the environment and human health. Since many nematicides and fumigants have been banned in the EU, there is a clear need for new nematicidal products (Chen et al., 2020).

Various alternatives to chemical PPPs have been proposed. The first possible alternative is the use of biopesticides, including PPPs from botanical origin (reviewed in Ntalli and Caboni, 2012; Chen and Song, 2021). Another option is to target host resistance instead of directly targeting the PPN. This can be achieved through resistance breeding (Milligan et al., 1998), genetic modification (Dutta et al., 2015), or ‘induced resistance’. Induced resistance (IR) involves the activation and priming of the immune system of the plant by an external stimulus (Mauch-Mani et al., 2016; De Kesel et al., 2021). Defense mechanisms will be activated faster and more intensively upon pathogen attack compared to naive plants (Mauch-Mani et al., 2016; De Kesel et al., 2021). For example, Benzothiadiazole (BTH), a salicylic acid-analogue (Melillo et al., 2014) is a successful resistance inducer against *M. incognita* in tomato. IR is a novel way of protecting plants from pathogen attack that has also been proven to be effective in the field (reviewed in Desmedt et al., 2021b).

In this work, we report on a new nematode management product consisting of ingredients of natural origin that combines nematicidal and resistance-inducing activities. A thorough literature study was done to select candidate bio-active compounds. The selected active ingredients include geraniol, rosemary and garlic extract, salicylic acid (SA), ascorbic acid (AA), and chitosan. A vegetable oil mixture consisting of sunflower, olive, and linseed oil was also added, which has both a nematicidal activity and formulation properties. The optimized mixture, together with emulsifiers and stabilizers, will henceforth be called ‘Product X’. The aim of this study was to examine how nematodes and plants react to this mixture. We investigated the direct nematicidal effect of Product X on *Meloidogyne incognita in vitro* and *in planta* in tomato (*Solanum lycopersicum)*. Moreover, the response of tomato plants to Product X treatment was examined at a transcriptional and biochemical level. Finally, we investigated the long-term effect of Product X application on tomato growth and development.

## 2 Material and methods

### 2.1 Growing tomato plants

Tomato (*Solanum lycopersicum* L. ‘Moneymaker’) seeds were sterilized before germination. Seeds were incubated for 2 minutes in 70% ethanol (Chemlab) after which they were incubated for 12 minutes in a 5% bleach (Guest Medical) solution with 0.0002 v% Tween20 (Sigma Aldrich) and rinsed 5 times with sterile H_2_O. After sterilization, seeds were put in moist potting soil (Structural Universal Type 1, Snebbout) and incubated in a growth room at a temperature of 24.5°C and light/dark regime of 16h/8h respectively. Seeds were incubated for 7 days before transplanting them to a sand:soil (1:1 v/v) mixture.

### 2.2 Culturing *Meloidogyne incognita*

Seeds of tomato plants were sterilized and germinated as described above. Plants of one month of age were inoculated with approximately 1000 *Meloidogyne incognita* J2 per pot containing three plants. These J2 originate from an in-house culture, initially provided by Shahid Siddique. Watering with a fertilizing solution (1 g/L Soluplant NPK 19–8-16 + 4 MgO + ME (micro-elements), Haifa Chemicals) happened twice per week depending on the age of the plants (200 ml – 750 ml per pot), until their harvest at 4 to 6 months after inoculation. At harvest, the required amount of galled roots (depending on the number of J2 needed per experiment) were separated from the potting soil and put on a sieve (200 µm pore diameter) covered with moist paper tissues (Tork). This sieve was put in a tray with tap water, allowing the nematodes to migrate from the roots through the sieve into the water. After 3 days, nematodes were collected by sieving the collection water over a sieve with pore diameter of 20 µm (Whitehead and Hemming, 1965). The number of nematodes in this solution was counted using a light microscope (Leica S8 APO, Leica microsystems).

### 2.3 Composition of Product X

Product X contains several components as listed in **Table 1**. Per compound, a weight percentage and a concentration in mg/L and mM (if possible) is displayed. The oil mixture makes up 20.5 wt% of Product X and consists of 10.3 wt% linseed oil, 67.1 wt% sunflower oil and 22.6 wt% olive oil.

**Table 1:** Composition of active ingredients of Product X in weight percentage, mg/L and – if possible – mM.

Active ingredient	Weight %	mg/L	mM	Manufacturer
**Geraniol**	**22.1**	**450.8**	**2.92**	**Sigma Aldrich – Merck group**
**Garlic extract**	**3.1**	**63.2**	**NA**	**Shaanxi Senlang Biochemical Co., LTD**
**Rosemary extract**	**1.4**	**28.6**	**NA**	**Sigma Aldrich – Merck group**
**Oil mixture**	**20.5**	**418.2**	**NA**	
** Linseed oil**	**10.3**	**43.1**	**NA**	**Vandeputte Group s.a.**
** Sunflower oil**	**67.1**	**280.6**	**NA**	**Group Vandamme NV**
** Olive oil**	**22.6**	**94.5**	**NA**	**Bertolli s.a.**
**Salicylic acid**	**1.0**	**20.4**	**0.15**	**Sigma Aldrich – Merck group**
**Ascorbic acid**	**3.1**	**63.2**	**0.36**	**Sigma Aldrich – Merck group**
**Chitosan**	**2.3**	**46.9**	**NA**	**Qingdao BZ oigo Biotech Co., LTD**

For complex mixtures no molecular weight is known, hence the concentration in mM cannot be calculated (indicated with NA – not available). The oil mixture present in Product X consists of linseed, sunflower and olive oil at a wt% of 10.3, 67.1 and 22.6, respectively. Emulgators and stabilizers used in the final formulation of Product X are not mentioned.

### 2.4 Direct nematicidal testing

The direct nematicidal effect of Product X was evaluated *in vitro* by incubating 50 *M. incognita* J2 in 500 µL of 0.2 v% Product X for 24 h or 48 h (**Supplementary Figure 1A**). As a negative control, nematodes were incubated in sterile tap water. As a positive control, 0.2 v% of Vertimec (active ingredient: abamectin) was used (Li et al., 2018). Nematodes were kept at room temperature and were counted at 24 h and 48 h using a stereo microscope. Nematodes were recorded as dead if they did not respond to prodding with a small dissection needle. For every treatment, 6 technical replicates were included. The experiment was repeated twice independently. Mortality was calculated relative to the negative control using the Schneider-Orelli equation (Schneider-Orelli, 1947).

### 2.5 Infection experiments

Plants were grown as described above. Two weeks after transplant (BBCH 103), tomato roots were treated with 20 mL of 0.2 v% Product X, 20 mL of water (negative control) or 20 mL of 0.2 v% Vertimec (positive control) (**Supplementary Figure 1B**). Per treatment, 8 plants were included. One or 3 day(s) after treatment, 250 J2 (*M. incognita*) per plant were inoculated on the plant roots. During their growth, plants were supplemented 3 times per week with 30 mL of a 1 g/L tomato fertilizer solution (Soluplant NPK 19–8-16 + 4 MgO + ME, Haifa Chemicals). Plants were harvested 28 days post inoculation: shoots were cut, dried (65°C for four days) and weighed, and roots were cleaned and stored in a 1:1 (v:v) ethanol:glycerol solution before galls were counted for every root system. After counting, roots were cleaned thoroughly, dried (65°C for four days) and weighed to determine root dry weight. The experiment was independently repeated two times.

### 2.6 mRNA-sequencing

Plants were grown as described above. After growing for two weeks in a soil:sand mixture, roots were treated with 20 mL of a 0.2 v% Product X solution (**Supplementary Figure 1C**). Control plants (roots) were treated with an equal amount of water. Every treatment consisted of 3 biological replicates that were pools of 4 individual plants. Plants were harvested 24 h after treatment, roots were washed to remove substrate and they were flash frozen in liquid N_2_. Samples were ground using a liquid nitrogen-chilled pestle and mortar, after which RNA was extracted from 100 mg of ground root tissue (RNeasy Plant Mini kit - Qiagen) with an extra sonication step after adding the RLT buffer (3 times 10 seconds) (Ghaemi et al., 2020). After extraction, RNA was washed to improve purity using ethanol-sodium acetate precipitation. Briefly, 20 µL extracted RNA, 3 µL 2 M sodium acetate (Sigma Aldrich) and 50 µL of absolute ethanol (Chemlab) were mixed and stored at -20°C overnight. Samples were centrifuged (Eppendorf-Centrifuge 5417R) for 20 min at 4°C at 19930 g. The supernatant was discarded and 200 µL of 75% absolute ethanol in RNAse free water was added. After centrifuging for 5 minutes at 4°C at full speed and removal of the supernatant, the pellet was dried for 15 min at 37°C. The pellet was redissolved in 20 µL RNAse free water. Quantity and purity were re-assessed using NanoDrop 2000 (ThermoFisher Scientific).

Prior to sequencing of complete mRNA of the samples, an RNA library was prepared using QuantSeq 3’ mRNA-Seq Library Prep Kit (Lexogen). Library quality was assessed using an Agilent Bioanalyzer 2100. RNA was sequenced using an IlluminaNextSeq 500. Library preparation and sequencing was performed by the NXTGNT facility (Ghent University, Belgium) (Ghaemi et al., 2020).

Analysis of RNA sequencing data starts with quality control of raw reads using FastQC (version 0.11.8) and Trimmomatic softwares (version 0.38; Window size = 4:30). Using STAR software (outFilterMultimapNmax = 1; outSAMtype = BAM SortedByCoordinate), trimmed reads were mapped onto the tomato reference genome (ITAG 4.0). Employing the GenomicAlignments (version 1.36.0) and DESeq2 (version 1.40.2) packages implemented in the Microsoft open R software (version 4.3) for quantifying read numbers (SummarizeOverlaps function) and differential expression analysis (DESeq function), respectively, differentially expressed genes (DEGs) were identified (FDR-adjusted p-value < 0.10). Exploratory data visualization was done via principal component analysis (PCA) using the rlog – for log2 transformation – and ggplot – for plotting – functions (DESeq2 and ggplot2 packages respectively). The volcano plot was produced using the R-package EnhancedVolcano (version 1.18.0). Gene ontology analysis was done using PLAZA 5.0 (Ghaemi et al., 2020) and g:Profiler (Raudvere et al., 2019).

### 2.7 Hormone measurements

Plants were grown as described above. After growing for two weeks in a soil:sand mixture, roots were treated with 20 mL of a 0.2 v% Product X solution (**Supplementary Figure 1D**). The control plants were water-treated. Every treatment consisted of 5 pools of tomato plants, with every pool containing material of 4 separate plants. Plants were harvested 24 h, 48 h or 72 h after treatment, and shoots and roots were crushed separately in liquid N_2_ until a fine grounded powder was obtained. Phytohormone extraction and analysis was performed according to Haeck et al (Haeck et al., 2018). Therefore, 100 mg of homogenized plant material (5 biological replicates per treatment, each biological replicate consists of 4 individual plants) was weighed in a 12 mL polypropylene tube (Greiner Bio-One International B.V.B.A.), and 5 mL of cold extraction buffer (approximately 4°C) was added. This buffer consisted of 75:20:5 (*v*/*v*/*v*) methanol, ultrapure water, and formic acid (Sigma Aldrich). Thereafter, the sample was vortexed (Kisker Biotech GmbH & Co. KG, via BaseClear Labproducts) for 30 s until all plant material was homogenized into the extraction solvent, after which they were shaken on ice for approximately 1 h. Subsequently, the samples were stored at − 80°C overnight. After the cold extraction, 4 mL of supernatant was transferred to a 30 kDa Amicon^®^ Ultra centrifugal filter unit (Merck Millipore), which was centrifuged for 30 min at 3900 rpm and 4°C in a SW9 R centrifuge (Froilabo). Afterwards, 2.5 mL of the purified extract was dried under N_2_ at 10°C by means of a Turbovap^®^ LV automated concentration evaporator (Biotage) in a silanized test tube. The extract was then redissolved with 0.5 mL methanol/water (20:80, *v*/*v*) with 0.1% formic acid, establishing a 5-fold concentration. This final extract was vortexed for 1 min and centrifuged for 2 min at 1000 rpm (EBA20, Andreas Hettich GmbH & Co.KG). The sample was then transferred to a HPLC-vial, and analyzed using an ultra-high performance liquid chromatography system, coupled to a Q-Exactive™ bench top MS/HRMS quadrupole-Orbitrap mass spectrometer, equipped with a heated electrospray ionization (HESI) source, and operating in both the positive or negative mode (Haeck et al., 2018). Xcalibur™ and TraceFinder (version 4.1) software (Thermo Scientific) were used for data processing, targeted plant hormone screening and quantification.

### 2.8 Peroxidase assay

Plants were grown as described above. After treating tomato roots with 20 mL of 0.2 v% Product X or 20 mL water for negative control plants, plants were harvested at 1, 2 and 3 days after treatment (**Supplementary Figure 1E**). For 1 day post treatment (dpt), every treatment consisted of 5 pools of tomato plants with every pool containing material of 4 separate plants. For 2 and 3 dpt, every treatment consisted of 10 pools of tomato plants with every pool containing material of 3 separate plants. Plant roots were crushed in liquid N_2_ and ground into a fine powder. Based on the protocol of MacAdam et al. (1992), using a photospectral method, 50 mg of crushed material was used to determine peroxidase levels per sample. First, an extraction buffer that consisted of 100 mM potassium buffer (K_2_HPO_4_ and KH_2_PO_4_; pH = 6, VWR), 0.8 M KCl (Sigma Aldrich) and 80 mg/mL polyvinylpolypyrrolidone (Sigma Aldrich) was made. Second, the assay buffer consisting of 100 mM potassium phosphate buffer (K_2_HPO_4_ and KH_2_PO_4_, pH = 6.0, VWR), 0.4 mM hydrogen peroxide (Sigma Aldrich) and 3.3 mM guaiacol (Sigma Aldrich) was prepared. The sample was suspended in 8 µL extraction buffer/mg sample, vortexed and centrifuged at 19930 g for 10 minutes at 4°C (Eppendorf-Centrifuge 5417R). Further steps were performed on ice. Ten µL of extract was transferred to a cuvette (disposable polystyrene cuvette, VWR) and 990 µL of assay buffer was added. After pipetting and measuring baseline absorbance at 436 nm, absorbance was measured at 30 s intervals for 3 minutes (VWR UV1600P).

Next, the total protein concentration in the sample was evaluated using a Bradford assay. In a 96-well plate, a combination of 20 µL extract and 180 µL of 1:5 diluted Bradford (Sigma Aldrich) reagent was measured at 595 nm after a 30 minute period of incubation at room temperature in the dark. Protein concentration can be calculated as the difference between the absorbance of the sample and the absorbance of the blank divided by 0.0014 (MacAdam et al., 1992). The peroxidase activity was then normalized by the total protein concentration per sample.

### 2.9 Malondialdehyde measurements

Plants were grown as described above. Every treatment consisted of 5 pools of tomato plants with every pool containing material of 4 separate plants (**Supplementary Figure 1D**). Plant material was ground in liquid nitrogen and 100 mg was used for executing malondialdehyde measurements. Ten µL/mg material of cold 5% w/v trichloroacetic acid in distilled water was added to each sample. After briefly vortexing, samples were centrifuged for 15 min at 13 000 rpm and 4°C. The supernatant was divided in 2 aliquots of 250 µL. After making a 2% w/v butylated hydroxytoluene in ethanol solution, 0.01% v/v of this solution was added to 20% w/v trichloroacetic acid in distilled water. 250 µL of this solution was added to one of the aliquots of sample supernatant. To the other aliquot, the same solution was added with the addition of 0.65% w/v thiobarbituric acid. Samples were heated in a hot water bath (95°C) for 30 minutes and then placed on ice to halt the reaction. After centrifuging at 4°C for 10 min, 100 µL of each sample was added to a 96 well plate and the malondialdehyde content was determined using a plate reader at 440, 532 and 600 nm and the formulas listed in Hodges et al (Hodges et al., 1999).

### 2.10 Long-term administration of Product X

The roots of two infected tomato plants (as described above) were cut and mixed with a potting soil and sand mixture (2:1, v/v). This mixture was moistened with tap water and incubated for 2 days at 25°C. The inoculated soil was treated with 500 mL water or with 500 mL 0.2 v% of Product X (**Supplementary Figure 1B**). After incubating for another 3 days at 25°C, 5 week old tomato plants were planted in the inoculated and treated soil. Per treatment, 8 plants were used. Plants were treated 7 times with 50 mL Product X or water as a control treatment with 2 week intervals. After 4 months, plants were harvested. The amount of tomatoes was counted, the biomass of plants was measured, and the efficacy of Product X treatment was assessed by scoring root galling using the Zeck scale (Zeck, 1971b). This experiment was independently repeated two times.

### 2.11 Statistical analysis

All data-analyses (statistical testing and graphical visualization) were done using Microsoft Open R software (version 4.3). Statistical tests were selected from the ‘stats’ package, while graphics were made using the ‘ggplot2’ package. P-values lower than 0.05 were regarded as statistically significant. Data-analysis of transcriptome data was already described earlier. Other data were first checked for normality with the Shapiro-Wilk test. Homoscedasticity of the data was checked using diagnostic plots. Both conditions were met for short-term infection experiments testing of Product X, student’s t-tests were performed between control and Product X-treated plants. Data of nematicidal assays (*in vitro* testing) of Product X were assessed using a generalized linear binomial model (GLM), combined with a Tukey range test. Differences between all treatments (water-treatment, Product X-treatment and Vertimec-treatment) were evaluated.

Data that did not adhere to normality or homoscedasticity were subjected to non-parametric testing. Data of hormone measurements, peroxidase and MDA-assays and long-term effects on yield were checked for significance by applying a Wilcoxon-Mann-Whitney test. Differences in median between values rendered by control-treated plants and Product X-treated plants were assessed.

## 3 Results

### 3.1 Product X has a direct nematicidal effect against root-knot nematodes and protects tomato plants from infection

**Figure 1:**
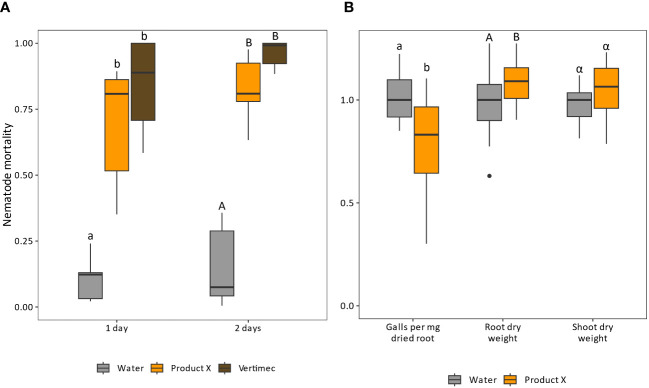
**(A)** Efficacy of Product X as a direct nematicidal agent. Vertimec (commercial formulation of abamectin) was used as a positive control. Product X as well as Vertimec were used at 0.2 v%. Nematode mortality was corrected to the negative control (not shown) using the Schneider-Orelli equation (Schneider-Orelli, 1947). Two independent repeats were performed. (n = 2x6). **(B)** Normalized gall number, root and shoot dry weight of plants inoculated with *M. incognita* at one day post treatment with Product X or water. Two independent repeats were performed. (n=2x8).

*In vitro* nematicidal potential of Product X was tested against *Meloidogyne incognita*. Exposure to Product X leads on average to 70.0 or 82.3% mortality of *M. incognita* after 1 day or 2 days, respectively (p-values < 0.001) (**Figure 1A**).

Next, the product was tested for its potential as nematicidal product in a pot trial with *Solanum lycopersicum* infected by *M. incognita*. Plant roots were treated with water or Product X, and inoculated with *M. incognita* juveniles 1 or 3 days later (**Figure 1B** and **Supplementary Figure 1**, respectively). After one growth cycle of the nematodes (28 days), root galls were counted and root and shoot dry weight were assessed. When inoculation was executed 1 dpt (dpt; **Figure 1B**), the number of galls per mg dried root tissue was significantly lower compared to water-treated plants (p-value = 0.0063). An average increase of 10% in root dry weight was observed compared to water-treated plants (p-value = 0.044), while the shoot dry weight of Product X-treated plants was not significantly affected (p-value = 0.21). When inoculation was done at 3 dpt (**Supplementary Figure 1**), a significant reduction in galls per mg of dried root was observed (p = 0.011), while no differences in root and shoot dry weight were detected (p-values = 0.60 and 0.76, respectively).

### 3.2 Product X induces transcriptional changes related to induced resistance in tomato at one dpt

**Figure 2:**
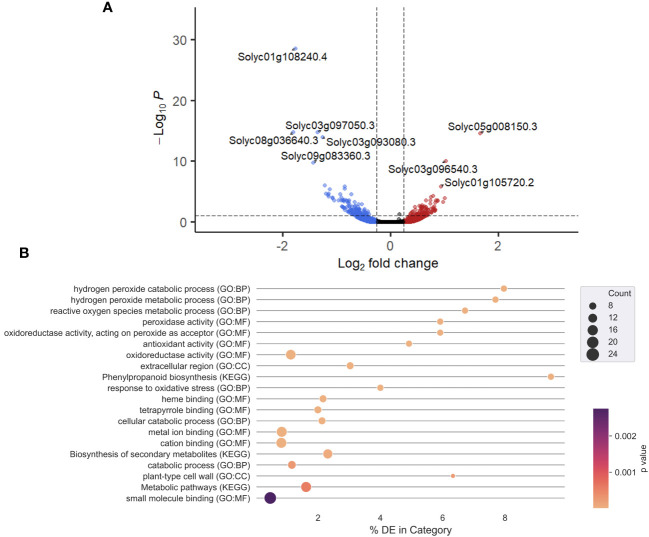
**(A)** Volcano plot of gene expression in roots treated with Product X. Horizontal line indicates threshold of a p-adjusted value higher than 0.10, while vertical lines indicate Log2FC of more than 0.25 (right, red) and lower than -0.25 (left, blue). Every point represents one gene. Upper left quadrant indicates genes with a p-adjusted value < 0.10 and Log2FC < -0.25, while the upper right quadrant indicates genes with a p-adjusted value < 0.10 and Log2FC > 0.25. Genes with the most extreme p-adjusted values are labeled. Their function is described in **Table 2**. **(B)** GO-analysis of significantly upregulated genes in roots treated with Product X. Top 20 gene ontology terms are displayed. Lighter colors indicate lower p-values, the size of the dots corresponds to the number of genes present in each category.

Product X contains ingredients such as salicylic acid that could potentially elicit induced resistance in treated plants. An mRNA-sequencing experiment was performed to evaluate this hypothesis, by monitoring gene expression in roots at 1 dpt. An exploratory principal component analysis (PCA) (**Supplementary Figure 3**) clearly separated water-treated and Product X-treated samples. A volcano plot (**Figure 2A**) reveals the general transcriptional response of all detected tomato genes in response to Product X treatment. Genes were considered as differentially expressed in response to Product X if the Log2FC was below 0.25 or above -0.25 and the adjusted p-values was below 0.10. An overview of all DEGs can be found in the **Supplementary Information**. Gene ontology and KEGG enrichment analyses were performed on up- or downregulated DEGs (significance level of 0.10) using g:Profiler (**Figure 2B**) (Raudvere et al., 2019). A complete overview of GO-terms in significantly up- and downregulated genes can be found in **Supplementary Information** (**Supplementary Table 1** and **Supplementary Figure 4**). GO terms such as *Hydrogen peroxide catabolic/metabolic process*, *Response to oxidative stress*, *Reactive oxygen species metabolic process* and *Peroxidase activity* are significantly enriched in the list of highly induced genes, indicating that ROS metabolism is significantly altered in roots of Product X-treated tomato plants.

Next, a more in-depth analysis of up- and downregulated genes was performed, focusing on ROS metabolism and phytohormone pathways, well-known hallmarks of induced resistance (De Kesel et al., 2021) (**Table 2**). Several peroxidase-encoding genes were upregulated, as the GO-analysis already suggested. Most genes associated with phytohormone signaling and/or biosynthesis were downregulated. Interestingly, an abscisic acid 8’-hydroxylase CYP707A2 (*Solyc08g005610.3*) was downregulated, indicating that the abscisic acid (ABA) level in roots treated with Product X might be elevated.

**Table 2:** A selection of differentially expressed genes (DEGs) related to ROS-metabolism and phytohormones.

Gene ID	Function	Log2FC
**Solyc08g036640.3**	**JAZ9**	**-2.75**
**Solyc01g108240.4**	**Ethylene Response Factor D3**	**-2.05**
**Solyc09g083360.3**	**Basic helix-loop-helix protein – DNA binding**	**-2.02**
**Solyc04g079730.1**	**Allene oxide synthase 1**	**-1.81**
**Solyc03g097050.3**	**Cellulose synthase-like protein**	**-1.59**
**Solyc03g093080.3**	**Xyloglucan endotransglucosylase/hydrolase 9**	**-1.46**
**Solyc08g005610.3**	**Abscisic acid 8’-hydroxylase CYP707A2 (ABA degradation)**	**-1.33**
**Solyc03g095770.3**	**WRKY70**	**-0.78**
**Solyc03g122340.3**	**lipoxygenase D (wound and JA-response)**	**-0.76**
**Solyc08g077020.1**	**Small auxin upregulated RNA 79**	**-0.69**
**Solyc05g046010.4**	**Peroxidase**	**0.49**
**Solyc06g051360.3**	**Gibberellin 2-beta-dioxygenase 1**	**0.56**
**Solyc07g047740.3**	**Peroxidase**	**0.57**
**Solyc01g015080.3**	**Peroxidase**	**0.58**
**Solyc02g092580.3**	**Peroxidase**	**0.59**
**Solyc01g108320.3**	**Peroxidase**	**0.72**
**Solyc03g080150.3**	**Peroxidase**	**0.76**
**Solyc02g087070.4**	**Peroxidase**	**0.83**
**Solyc12g005790.2**	**Peroxidase**	**1.05**
**Solyc01g006310.3**	**Peroxidase**	**1.06**
**Solyc01g105720.2**	**Unknown function**	**1.12**
**Solyc03g096540.3**	**Wound/stress protein**	**1.17**
**Solyc05g008150.3**	**Metallocarboxypeptidase inhibitor**	**2.27**

DEGs were identified by comparing expression in roots of Product X-treated tomato plants versus control plants. The eight genes with the lowest p-adjusted values were also incorporated based on the Volcano plot (**Figure 2A**). DEGs were identified based on adjusted p-values < 0.10. Log2FC (Log-2 fold change) value indicates a Log2 transformed change in gene expression of Product X-treated roots versus water-treated roots. Negative values indicate downregulation, while positive values signify upregulation. A complete overview of DEGs can be found in **Supplementary Information**.

### 3.3 Phytohormone levels in roots and shoots are altered upon Product X application

Phytohormone measurements were performed at three different timepoints, i.e. at 1, 2 and 3 dpt with Product X or water (negative control) and in both roots and shoots (summarized in **Table 3** and in more detail in **Supplementary Figures 5–8**). For JA a transient decrease over time was observed in roots (p-values = 0.056 and 0.0079, respectively), but not in shoots. Similarly, a strong but transient decrease of SA was observed in root tissue at 1 and 2 dpt with Product X (both p-values = 0.0079), but no differences in shoot tissue. ABA level increased strongly in the roots at 1 and 2 dpt, but decreased after three days (p-values = 0.016, 0.0079 and 0.0079, respectively). In the shoots treated with Product X, an increase in ABA was observed at all three timepoints (p-values = 0.0079, 0.0079 and 0.016, respectively). For IAA, a decrease at 2 and 3 dpt was detected in root tissue (p-values = 0.0079 and 0.016).

**Table 3:** Summarized overview of fold changes in phytohormone levels in roots and shoots of Product X-treated plants compared to water-treated plants (negative control).

	Root			Shoot		
	1 dpt	2 dpt	3 dpt	1 dpt	2 dpt	3 dpt
**JA**	0.50	**0.33**	0.67	1.06	0.87	0.92
**SA**	**0.56**	**0.54**	1.00	0.98	1.30	1.16
**ABA**	**1.32**	**3.85**	**0.78**	**1.31**	**1.91**	**1.15**
**IAA**	0.87	**0.78**	**0.70**	NA	NA	1.30

Fold change was calculated as the quotient of medians of phytohormone levels of Product X-treated compared to water-treated material (shoot or root). A fold change > 1 means an increase in phytohormone level in Product X-treated plants versus water-treated plants. Similarly, a fold change < 1 means a decrease. Values in bold indicate a significant difference between medians of phytohormones in Product X-treated and water-treated plants. NA indicates that phytohormone levels were below the limit of detection. Actual hormone levels (ng.mg^-1^ fresh weight) and data visualization in figure format are represented in **Supplementary Information** (**Supplementary Figures 5–8**).

### 3.4 ROS-metabolism is altered by Product X treatment

**Figure 3:**
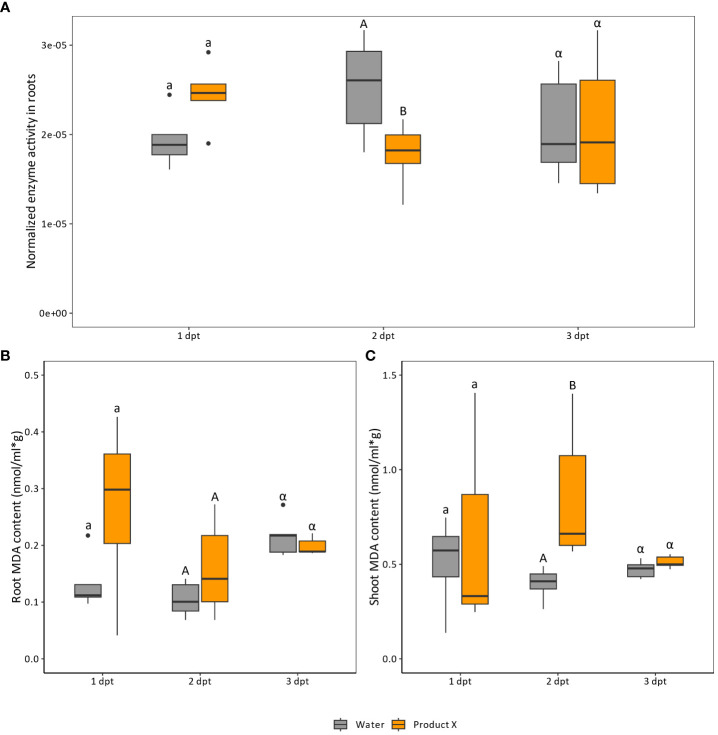
**(A)** Peroxidase levels in root material treated with Product X at one (n=5), two (n=10) and three (n=10) days post treatment. At one dpt 5 pools of 4 plants were used, while at two and three dpt 10 pools of 3 plants were assessed. **(B, C)** MDA content (nmol MDA/(ml*g fresh sample weight) in root **(B)** and shoots **(C)** in water-treated and Product X-treated plants. Water treatment was used as a negative control treatment. Each biological replicate consists of a pool of 4 plants. (n=5). Per timepoint comparisons were made between control and Product X-treated plants. Statistical significance is indicated with a/b for 1 dpt, A/B for 2 dpt and α/β for 3 dpt. Different letters indicate statistical significant comparisons.

Based on the sequencing data and the composition of Product X, it was hypothesized that ROS-metabolism could be altered upon Product X treatment. To confirm this hypothesis, peroxidase levels in the roots (**Figure 3A**) and malondialdehyde (MDA) levels, in the roots (**Figure 3B**) and shoots (**Figure 3C**) of treated plants were measured at 1, 2 and 3 day(s) post treatment.

For peroxidase levels in roots, an increase at 1 dpt followed by a decrease at 2 dpt was observed (p-values = 0.055 and 0.0015). However, at 3 dpt, the effect leveled out, and both Product X and water treated plants displayed the same peroxidase activity in the roots (p-value = 0.58).

MDA is a product of lipid peroxidation and is considered as an approximation for endogenous ROS levels and oxidative stress in plants (Morales and Munné-Bosch, 2019). In roots, no significant difference in MDA was detected between Product X-treated and water-treated plants both at 1 and 2 dpt (p-values = 0.28 and 0.34 respectively). However, in shoots, an increase in MDA-levels in Product X-treated plants was observed at 2 dpt (p-value = 0.0079), while this was not the case for 1 dpt (p-value = 1.0). At 3 dpt, no significant changes were observed for both root and shoot MDA content.

### 3.5 No negative effects on plant growth and yield were observed upon treating plants with Product X throughout their growth season

**Figure 4:**
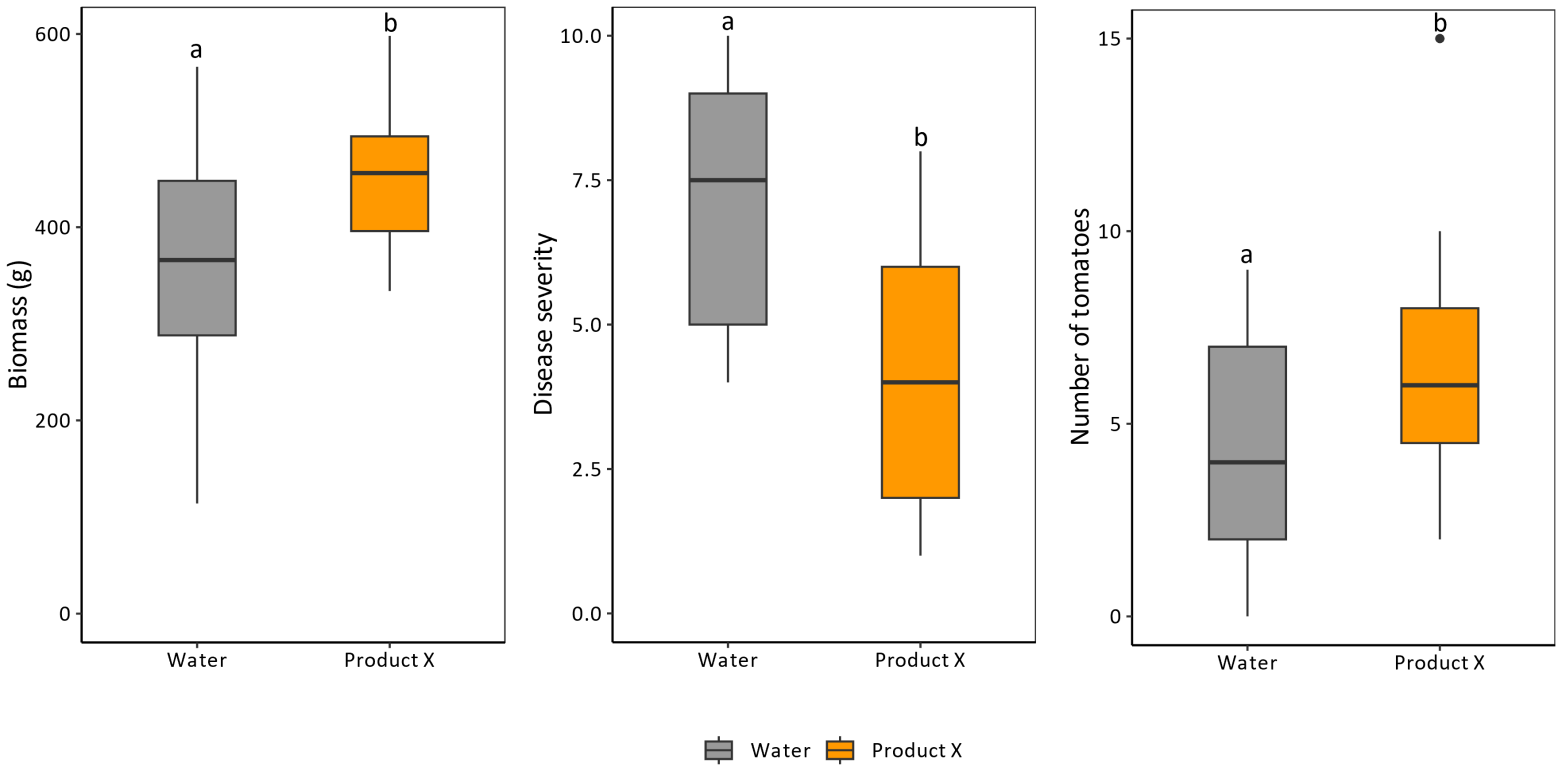
Effects of long-term application (8 times over a course of 4 months) of Product X on biomass production, nematode disease severity and number of tomatoes. Disease severity is expressed by the Zeck-scale which is an ordinal scale between 0 and 10, with 0 being no nematode infection (Zeck, 1971a). Two independent repeats were performed. (n = 2x8).

During prolonged administration (8 times over the course of 4 months) of Product X to tomato plants, no negative but rather positive effects were observed on growth or development (**Figure 4**). The biomass (shoot weight) and number of tomatoes of treated plants was significantly increased compared to control plants (p-values of 0.034 and 0.046, respectively). Additionally, Zeck-scale (Zeck, 1971a) evaluation of nematode infestation revealed a decrease of approximately 40% in treated plants compared to untreated plants (p-value = 0.0022). This could possibly explain the increase in biomass and number of tomatoes in treated plants (**Figure 4**).

## 4 Discussion and conclusions

### 4.1 Product X has a direct and indirect effect on *Meloidogyne incognita*

Product X was tested for its direct nematicidal activity against *Meloidogyne incognita*. After 48 h, nematode mortality amounted to over 80% (**Figure 1A**). Next to that, Product X reduces galling in tomato plants infected by *M. incognita* (**Figure 1B** and **Supplementary Figure 1**, **Figure 4**). Several components included in Product X have previously described *in vitro* and *in vivo* nematicidal properties, namely geraniol, garlic and rosemary extracts, salicylic acid and linseed oil. For every compound, the concentration reported in literature was here converted to the equivalent in mg/L (*between brackets and italics*) to ensure ease of comparison between studies.

First, geraniol was previously reported to be nematicidal against several nematodes and was included in Product X at a concentration of 451 mg/L. Geraniol belongs to the group of monoterpenes, which are produced by plants as secondary metabolites. Monoterpenes contain several well-known compounds such as geraniol, menthol and citral. Geraniol in particular has been attributed antitumor and antidiabetic activities (Lei et al., 2019), as well as broad-spectrum nematicidal activity. Tsao and Yu tested several monoterpenoid compounds against *Pratylenchus penetrans* and found an *in vitro* nematicidal activity of geraniol of 43% at a concentration of 250 µg/mL (*250 mg/L*) (Tsao and Yu, 2000). Antinematode activity against *M. incognita* was evaluated by Echeverrigaray et al. by investigating egg hatching ability and J2 mobility *in vitro*. Both parameters were reduced by more than 90% compared to the control treatment after 48 h, at a concentration of 500 mg/L geraniol (Echeverrigaray et al., 2010). Next to that, geraniol concentrations of 100 and 250 mg/kg substrate (*1996 and 4990 mg/L*, respectively), significantly reduced galling of tomatoes planted in nematode infected and geraniol treated substrate (Echeverrigaray et al., 2010). Similar studies were performed to evaluate the effect on *M. javanica* and *Ditylenchus dipsaci* (Nasiou and Giannakou, 2018; Stavropoulou et al., 2021). After 48 h, no nematode mobility could be observed for *M. javanica* at a geraniol concentration of 500 ppm (*499 mg/L*) (Nasiou and Giannakou, 2018). For *D. dipsaci*, a concentration of 2000 µL/l (*1778 mg/L*) rendered on average 40% nematode immobility after 48 h (Stavropoulou et al., 2021). The concentration of geraniol in Product X is 451 mg/L, which is in the range of other studies reported here. The observed activity is hence in line with other studies.

Second, an extract of garlic is included in Product X at a concentration of 63.2 mg/L. Nematicidal effects of garlic extracts have been reported at different timepoints and concentrations. D’Addabbo et al. found an *in vitro* mortality of 100% for *Xiphinema index* – the California dagger nematode – after 8 h of exposure at a concentration of 0.5 mL/L (*390 mg/L*) Nemguard, a commercially available garlic extract-based nematicide (D’Addabbo et al., 2023). The same concentration leads to 50% reduction of the *X. index* soil population (D’Addabbo et al., 2023). Similarly, a watery extract (10 g FW/100 mL H_2_O) of fresh garlic leaves reduces *M. incognita* viability *in vitro* by 22% and in soil by almost 60% (Abo-Elyousr et al., 2009). *M. incognita* galling was reduced by almost 60% under greenhouse conditions when the treatment was applied twice with a 20 day interval (Abo-Elyousr et al., 2009). However, when compared to the results of Abo-Elyousr et al., the *in vitro* effectivity of Product X is higher than that of pure garlic extract (Abo-Elyousr et al., 2009). The nematicidal activity of garlic extract has been attributed to diallyl polysulfide compounds (Block et al., 1993), bio-active molecules that are derived from sulfur-containing amino acids by enzymatic transformation (Block et al., 1993). For example, pure allicin displays 100% mortality against *M. incognita* after 48 h of *in vitro* exposure at a concentration of 12.5 mg/L (Block et al., 1993; Gupta and Sharmaj, 1993). Although similar results have been regularly reported in literature, small differences could be due to differences in concentrations (D’Addabbo et al., 2023), susceptibility of different nematodes (D’Addabbo et al., 2023), application methods (Abo-Elyousr et al., 2009), or the use of a pure active compound versus an extract (Gupta and Sharmaj, 1993).

Third, an essential oil derived from *Salvia rosmarinus* was included in Product X at a concentration of 28.6 mg/L. According to literature, the effect of *S. rosmarinus* is two-fold, i.e. both nematicidal and inducing plant resistance. During a two-year field trial, oil derived from *S. rosmarinus* was observed to protect *Pisum sativum* against *M. javanica* (Mattei et al., 2014), although only in one of the two trials. A concentration of 3% (*27 240 mg/L*) rendered approximately 50% reduction in gall number (Mattei et al., 2014). Essential oil extracted from *S. rosmarinus* at a concentration of 1.5 v% (*6.81 mg/L*) causes an *in vitro* nematicidal effect of 100% against *Meloidogyne* sp. after 50 h of exposure (Iler-Iler et al., 2017). The first study used a significantly higher amount of *S. rosmarinus* extract than the concentration in Product X, while the latter only tested the *in vitro* effect of the *S. rosmarinus* extract.

A fourth compound present in product X, salicylic acid (SA, 20.4 mg/L), has been reported to have nematicidal properties. *In vitro* SA had an LC50 value of 46 mg/L on *Meloidogyne incognita* (Wuyts et al., 2006). The low pH of this solution (3.0), could partly explain its nematicidal effect. However, the activity of SA as stimulus of induced resistance is well-known (Corina Vlot et al., 2009; Liu et al., 2019; Saleem et al., 2020).

Finally, a concentration of 1 mL/L (*930 mg/L*) linseed oil reduces the nematode population in pepper plants by 36–50% depending on the used cultivar (Eldeeb et al., 2022). Formulated linseed oil was tested against *M. incognita* infecting tomato plants and 26% less galls were observed at a dose of 0.3 mL formulated linseed oil/kg substrate (Radwan et al., 2007). Product X contains 43.1 mg/L linseed oil, which is remarkably lower compared to literature values. The addition of (linseed) oil has a dual function, combining nematicidal activity with positive effects on product stability.

Several components present in Product X have been reported to hold anti-nematode activity *in vitro* and/or *in vivo*. However, all these compounds were tested as standalone treatments in previous studies. This sometimes limits the concentration range in which a product can be used, because of issues of phytotoxicity (Echeverrigaray et al., 2010). Combining different components with similar activities can render an additive effect in the combined mixture, without exceeding phytotoxic concentrations for any individual compound, allowing to work with lower concentrations of active compounds in mixtures versus single molecules. Probably, geraniol, garlic extract, rosemary essential oil and linseed oil contribute to the anti-nematode activity observed in this work (**Figure 1A**).

### 4.2 Product X induces transcriptional and biochemical changes in roots and shoots of tomato

To investigate the hypothesis that plant defense mechanisms would be activated upon Product X treatment in tomato, transcriptional analyses linked with biochemical validation were performed.

An mRNA-sequencing experiment was performed on Product X-treated and water-treated plants to assess transcriptional changes. Principal component analysis (PCA) revealed a clear separation between Product X-treated and water-treated tomato plants (**Supplementary Figure 3**), indicating that transcriptomic changes occur in Product X-treated plants. Gene ontology enrichment analysis revealed significant enrichment of GO-terms related to ROS metabolism among the DEGs (**Figure 2B**). Appropriately timed and localized ROS bursts play an important role in plant defense (Balmer et al., 2015). Other hallmarks of plant defense activation include altered phytohormone homeostasis, cell wall reinforcement via (often ROS- and peroxidase-dependent) mechanisms such as callose deposition, oxidative cross-linking and lignification (Balmer et al., 2015; Liu et al., 2015, 2018).

Among the DEGs, a large group of genes is related to phytohormone- and ROS-metabolism. Genes related to phytohormones (JA, ethylene (ET), SA and auxin) are generally downregulated. However, an ABA hydroxylase, which is related to ABA degradation, is downregulated (**Table 2**). This was confirmed by phytohormone measurements in roots, where ABA levels increase at 1 dpt and rise further at 2 dpt with Product X (**Table 3**). At 3 dpt with Product X, root ABA levels decrease, probably because of a negative feedback mechanism. In shoots, ABA levels increase at all three timepoints (**Table 3**). Iriti and Faoro showed that ABA plays a role in chitosan-induced resistance against tobacco necrosis virus (Iriti and Faoro, 2008). ABA levels inside the plant increased three-fold upon application of chitosan (Iriti and Faoro, 2008). The observed endogenous ABA increase could be induced by the presence of chitosan in Product X.

ABA is commonly associated with responses to abiotic stress such as drought, salinity and cold (Finkelstein, 2013), but also plays an important but complex role in plant immunity that is highly dependent on concentration, pathosystem and environment (Mauch-Mani and Mauch, 2005). Based on the observed endogenous ABA increases in tomato roots, we hypothesize that Product X can also protect plants from abiotic stresses. Although not studied in this work, this could render new and exciting research opportunities.

Next to that, levels of JA and SA decrease strongly at 1 and 2 dpt in root tissue (**Table 3**). It has been shown by Nahar et al. that exogenously applied ABA can interfere with JA, SA and ET pathways increasing susceptibility of *Oryza sativa* for *Hirschmanniella oryzae* (Nahar et al., 2012). At 3 dpt, the levels of SA and JA have returned to basal levels in Product X treated roots. In shoots, no differences in JA or SA levels were detected at any time point (**Table 3**). Similarly, combined foliar application of COS-OGA – chitosan oligomers and pectin-derived oligogalacturonides – has been shown to induce resistance in rice against *Meloidogyne graminicola* independently of JA or SA (Singh et al., 2019). Instead, the phenylpropanoid pathway was a main player in the induced resistance (Singh et al., 2019). This was also the case in tomato where 0.3 mM (*49.8 mg/L*) piperonylic acid induced resistance against *Meloidogyne incognita* via the phenylpropanoid pathway, independent of JA- or SA-levels (Desmedt et al., 2021a). Similarly, the phenylpropanoid pathway may play a role in Product X-treated plants (**Figure 2B**). *Phenylalanine ammonia-lyase* (PAL) and *Cinnamoyl-COA reductase* encode important enzymes early in the phenylpropanoid pathway and, seeing that these genes are upregulated, this could have downstream effects in the phenylpropanoid pathway.

SA was strongly reduced in roots at 1 and 2 dpt with Product X (**Table 3**). It is important to note that SA is present in Product X at a concentration of 20.4 mg/L. This hormone has been implicated in several plant physiological processes including but not limited to flowering, disease resistance as well as tolerance to certain abiotic stresses (Corina Vlot et al., 2009; Liu et al., 2019; Rhaman et al., 2021; Barwal et al., 2023). SA plays a major role in Systemic Acquired Resistance (SAR), which is a form of induced resistance (De Kesel et al., 2021). In addition, SA affects the plant antioxidant system by regulating key enzymes such as dehydroascorbate reductase and glutathione reductase (Mustafa et al., 2018; Yan et al., 2018; Saleem et al., 2020). Reduction of SA levels in roots could be caused by a negative feedback loop that is activated because of the presence of SA in Product X.

Indole-3-acetic acid (IAA), the principal auxin, was less abundant in roots at 2 and 3 dpt (**Table 3**). IAA is elevated locally during nematode infection, and required for successful giant cell development (Oosterbeek et al., 2021). The lower endogenous IAA content in Product X treated roots could hamper nematode infection and/or alter their post-invasion development. Although IAA plays important roles in plant growth and development (Gomes and Scortecci, 2021), tomato plants do not seem to suffer from repeated Product X application, while on the contrary minor positive effects were observed (**Figures 1B**, **4**).

In this work, we observed an upregulation of genes encoding peroxidases in roots treated with Product X (**Table 2**). The disturbance of ROS homeostasis in treated plants could be linked to the presence of SA and AA, which both act as antioxidants (Cheng et al., 1996). Confirming mRNA-sequencing results, peroxidase levels were increased at 1 dpt in treated roots, while a decrease was observed at 2 dpt. At 3 dpt, levels of treated and untreated roots converged to the same level (**Figure 3A**). Next to that, MDA content, considered as a proxy for lipid peroxidation and linked to cell wall degradation, was determined in roots and shoots treated with Product X (**Figures 3B, C**). While no significant difference in MDA levels was observed in roots (**Figure 3B**), shoots contain significant higher MDA-levels at 2 dpt with Product X (**Figure 3C**). The lack of strong effect in MDA levels could be due to the presence of both SA and AA as antioxidants. Free ROS could also be used for cell wall modifications by the elevated peroxidase activity. This could explain why even though transcriptional and biochemical changes in peroxidase activity were observed, clear signs of oxidative stress were not observed in the roots of treated plants. Furthermore, it should be taken into account that when MDA levels are measured on complex plant mixtures, interfering agents such as carbohydrates or anthocyanins could be present (Morales and Munné-Bosch, 2019).

### 4.3 Product X shows no negative effects upon long-term application

Product X influences phytohormone homeostasis – SA and JA – and ROS metabolism which are both considered hallmarks of plant defense (De Kesel et al., 2021). However, influencing and/or activating pathways *in planta* can bring about a certain fitness cost that has a negative effect on plant growth and development (De Kesel et al., 2021). Next to that, geraniol and SA have been reported to cause phytotoxicity when used at high concentrations (>250 mg/kg and 276.24 mg/l respectively) (Echeverrigaray et al., 2010; Tripathi et al., 2019; Koo et al., 2020).

To assess whether this occurs at the concentrations used, Product X was repeatedly applied to infected tomato plants over a prolonged period of time. No negative effect on growth or development could be observed (**Figure 4**). On the contrary, plant biomass and number of tomatoes increased.

In this research, we demonstrate that Product X has a nematicidal effect on the root-knot nematode *M. incognita*. Next to that, transcriptional and biochemical analyses revealed that Product X influences phytohormone levels and ROS metabolism *in planta*. This suggests that Product X can activate plant defense mechanisms that help protect tomato against *M. incognita*. Furthermore, ABA was strongly induced in Product X-treated plants, with potential benefits for abiotic stress tolerance that should be investigated in future research. This work indicates that there is an additive effect of the compounds in Product X. Finally, no negative long-term effects of repeated product X administration on tomato growth and development were observed. This renders Product X an interesting candidate to be included in IPM strategies to control parasitic nematodes.

## Data availability statement

The datasets presented in this study can be found in online repositories. The names of the repository/repositories and accession number(s) can be found below: https://www.ncbi.nlm.nih.gov/, PRJNA1095548.

## Author contributions

ED: Conceptualization, Data curation, Formal Analysis, Funding acquisition, Investigation, Methodology, Project administration, Resources, Software, Validation, Visualization, Writing – original draft, Writing – review & editing. CS: Investigation, Writing – review & editing. SP: Conceptualization, Funding acquisition, Investigation, Methodology, Project administration, Resources, Supervision, Writing – review & editing. BS: Software, Writing – review & editing. KD: Investigation, Writing – review & editing. SM: Conceptualization, Funding acquisition, Investigation, Methodology, Project administration, Resources, Supervision, Writing – review & editing. TK: Conceptualization, Funding acquisition, Investigation, Methodology, Project administration, Resources, Supervision, Writing – review & editing.
