# Supplementary material for: A combination of plant-based compounds and extracts acts nematicidal and induces resistance against Meloidogyne incognita in tomato
Source: Front Plant Sci. 2024 Jul 4;15:1411825. doi: 10.3389/fpls.2024.1411825 (PMC11254767; doi:10.3389/fpls.2024.1411825)
Supplement: Supplementary file 1 [file DataSheet_1.docx]

Supplementary Material

**A combination of plant-based compounds and extracts acts nematicidal and induces resistance against Meloidogyne incognita in tomato.**

**Eva Degroote^1,2,3^, Chloë Schoorens^1^, Stefaan Pockelé^3^, Boris Stojilković^1^, Kristof Demeestere^4^, Sven Mangelinckx^2*^, Tina Kyndt^1*^**

*** Correspondence:**

Tina Kyndt

[tina.kyndt@ugent.be](mailto:tina.kyndt@ugent.be)

Sven Mangelinckx

[sven.mangelinckx@ugent.be](mailto:sven.mangelinckx@ugent.be)

# Supplementary Figures

## Visual representation of experimental design of experiments


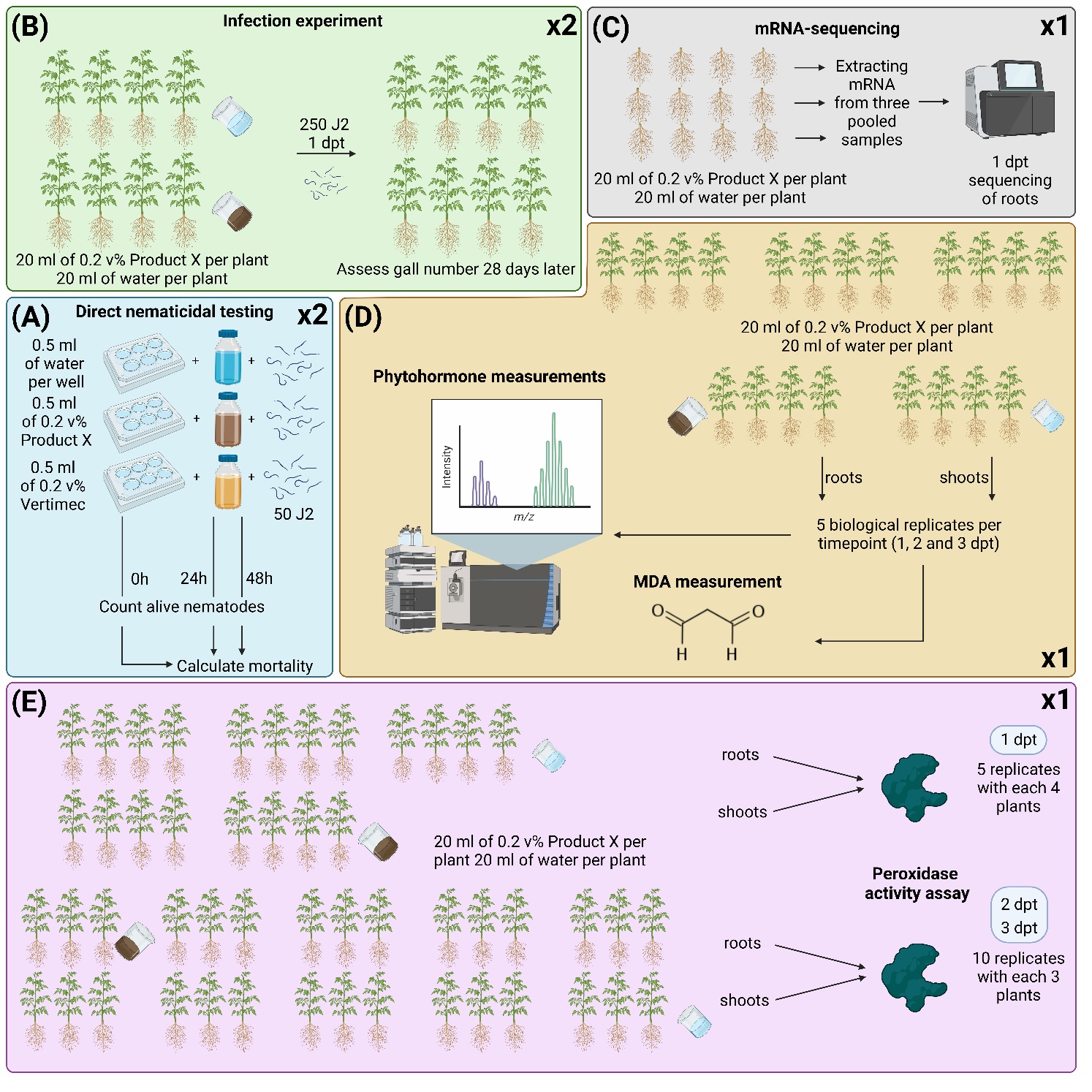


*Supplementary Figure 1 – Visual representation of experimental set-up. Treatments: light blue solution = water; dark brown solution = Product X. (A) Direct nematicidal testing was performed on six replicates per treatments. This experiment was independently repeated. (x2). (B) Infection experiments were performed twice with 8 plants per treatment for long and short term effects of Product X (x2). (C) mRNA-sequencing of root samples was performed on 3 pools of 4 plants (x1). (D) For both phytohormone and MDA measurements roots and shoots of 5 pools of 4 plants were used per timepoint (1,2 and 3 dpt) to perform the analyses (x1). (E) Peroxidase activity assays were performed on roots and shoots of 5 pools of 4 plants for 1 dpt and 10 pools of 3 plants for 2 and 3 dpt (x1).*

## Product X protects tomato against root-knot nematode infection at 3 days post treatment.

**
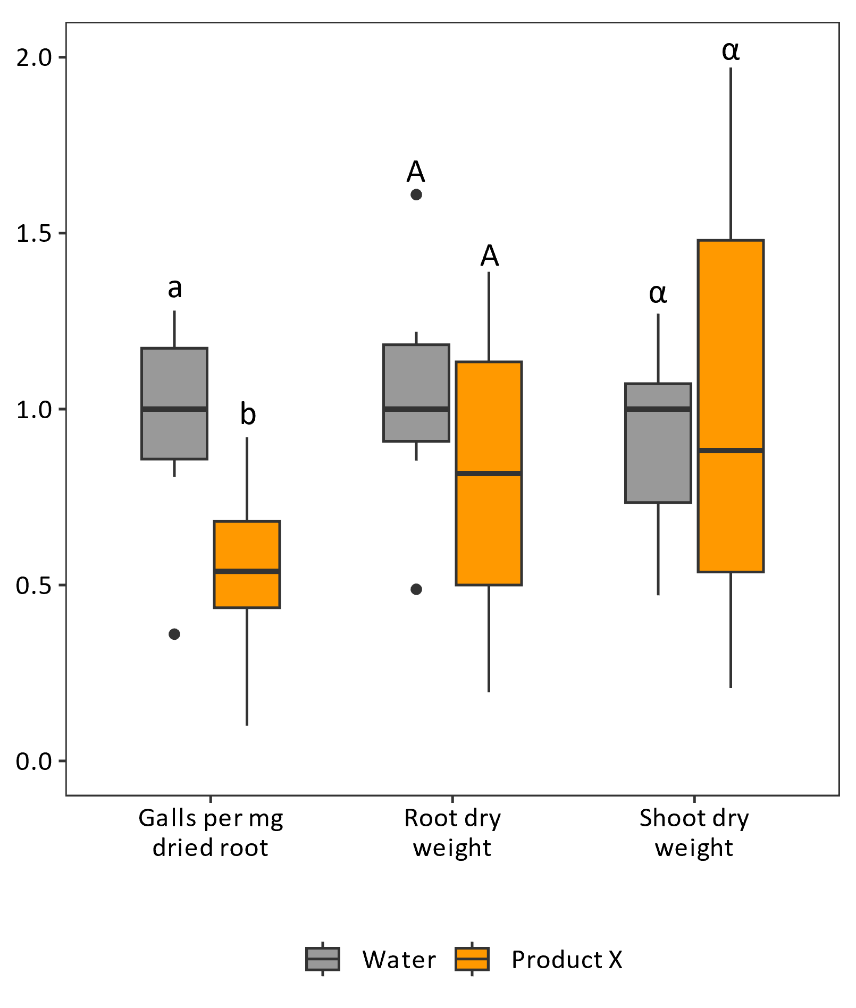
**

*Supplementary Figure 2 – Normalized gall number, root and shoot dry weight of plants inoculated with M. incognita at three days post treatment with product X or water. Gall number is significantly different from water treated plants (n = 8).*

## Principal component analysis of gene counts for Water- and Product X-treated genes.

*
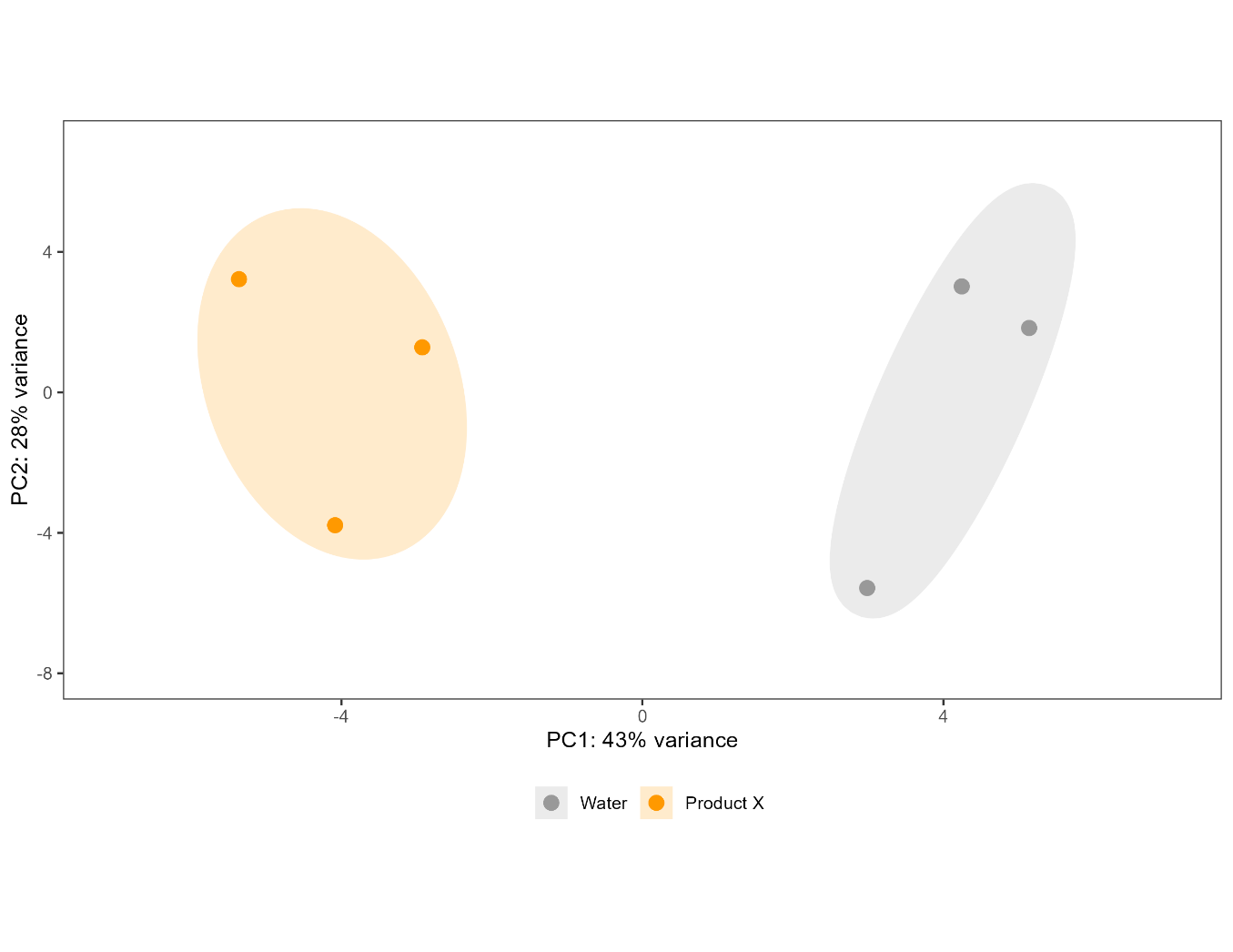
*

*Supplementary Figure 3 – PCA of gene counts for water-treated and product X-treated roots. Ellipses are 95% confidence contours (n = 3).*

## Overview of all GO-terms significantly enriched in downregulated DEGs in roots treated with Product X.


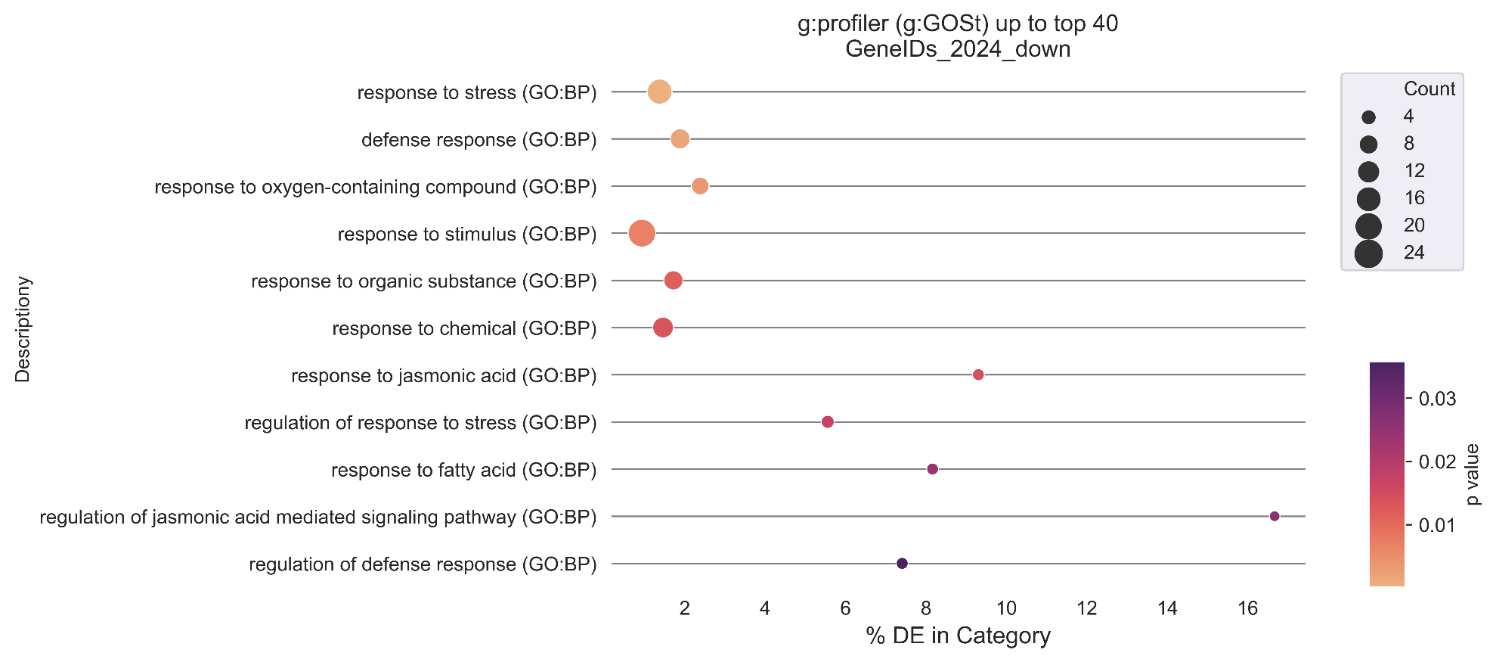


*Supplementary Figure 4 –* *GO-analysis of significantly downregulated genes in roots treated with product X. All gene ontology terms are displayed. Lighter colors indicate lower p-values, the size of the dots corresponds to the number of genes present in each category.*

## Quantification of phytohormone levels in Product X-treated plants

Figures included hereafter (Supplementary Figures 4-7) are an elaboration on Table 3 included in the main text. For every timepoint, the figures show the of phytohormone content in plant material (root or shoot) of tomato plants treated with water (negative control) or Product X.

**
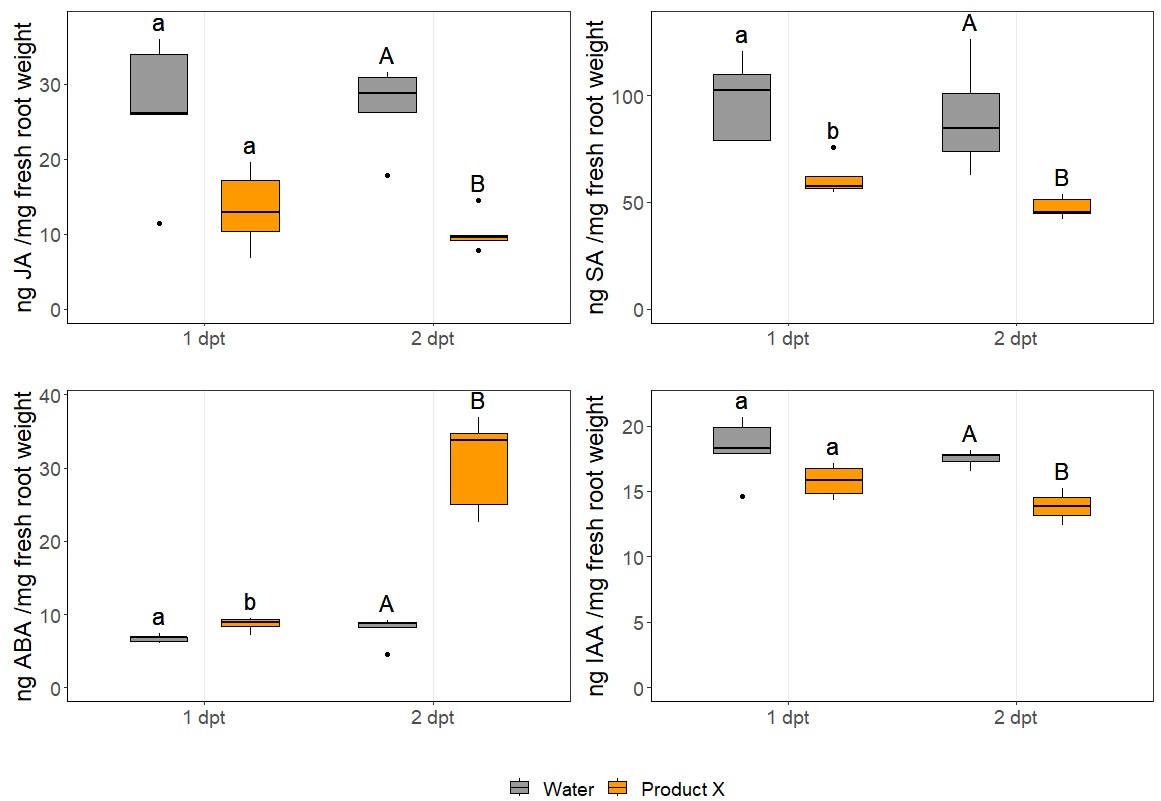
**

*Supplementary Figure 5 – Phytohormone levels at 1 and 2 day(s) post treatment (dpt) in root material of water-treated and product X-treated plants (n=5). Data of control and Product X-treated plants was compared per phytohormone and per timepoint. Per comparison, lower or capital letters were used to indicate significant differences.*

**
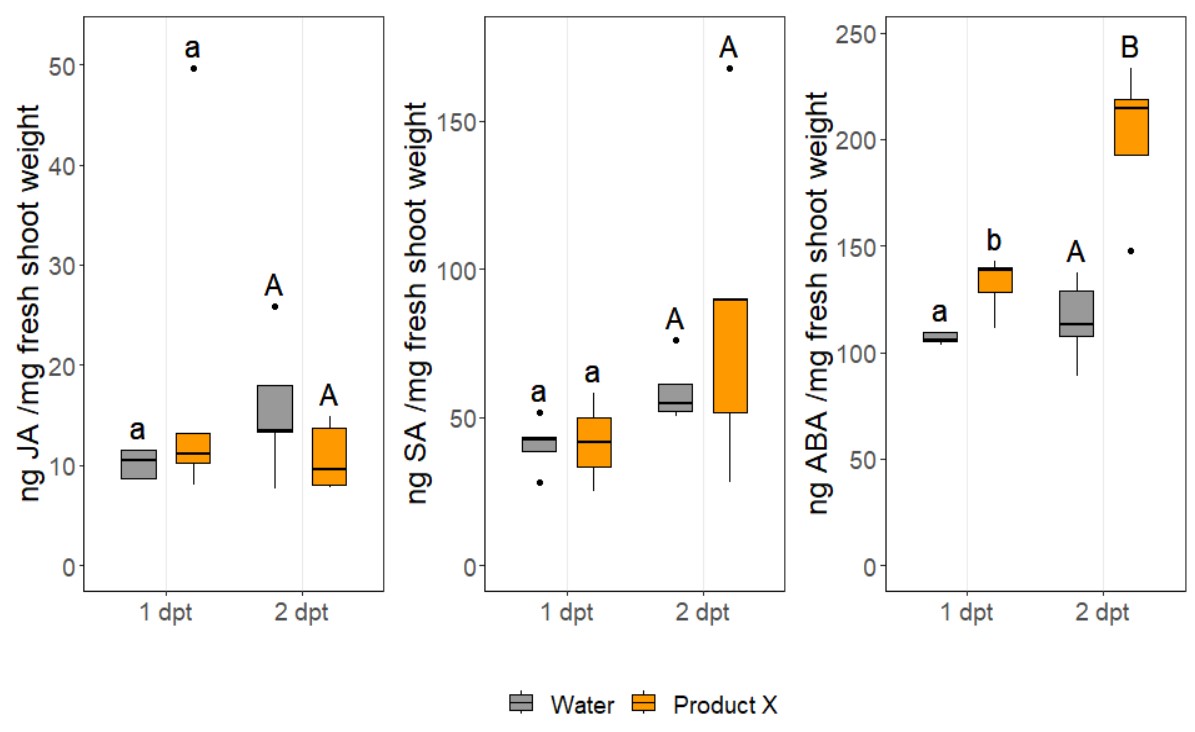
**

*Supplementary Figure 6 – Phytohormone levels at 1 and 2 day(s) post treatment (dpt) in shoot material of water and product X treated plants (N=5). Data of control and Product X treated plants was compared per phytohormone and per timepoint. Per comparison lower or capital letters were used to indicate significant differences.*

**
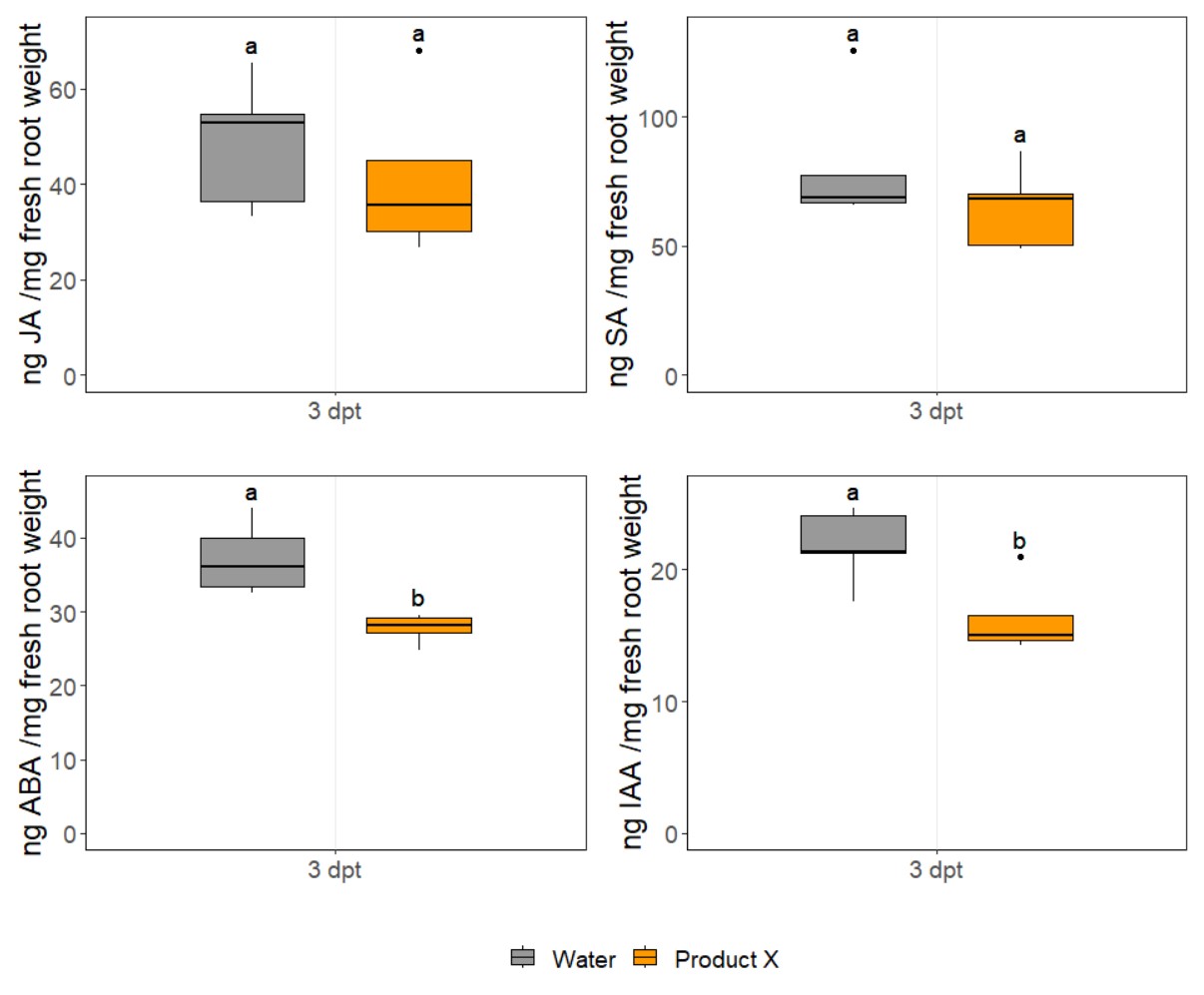
**

*Supplementary Figure 7– Phytohormone levels at 3 days post treatment (dpt) in root material of water and product X treated plants (N=5). Data of control and Product X treated plants was compared per phytohormone.*

**
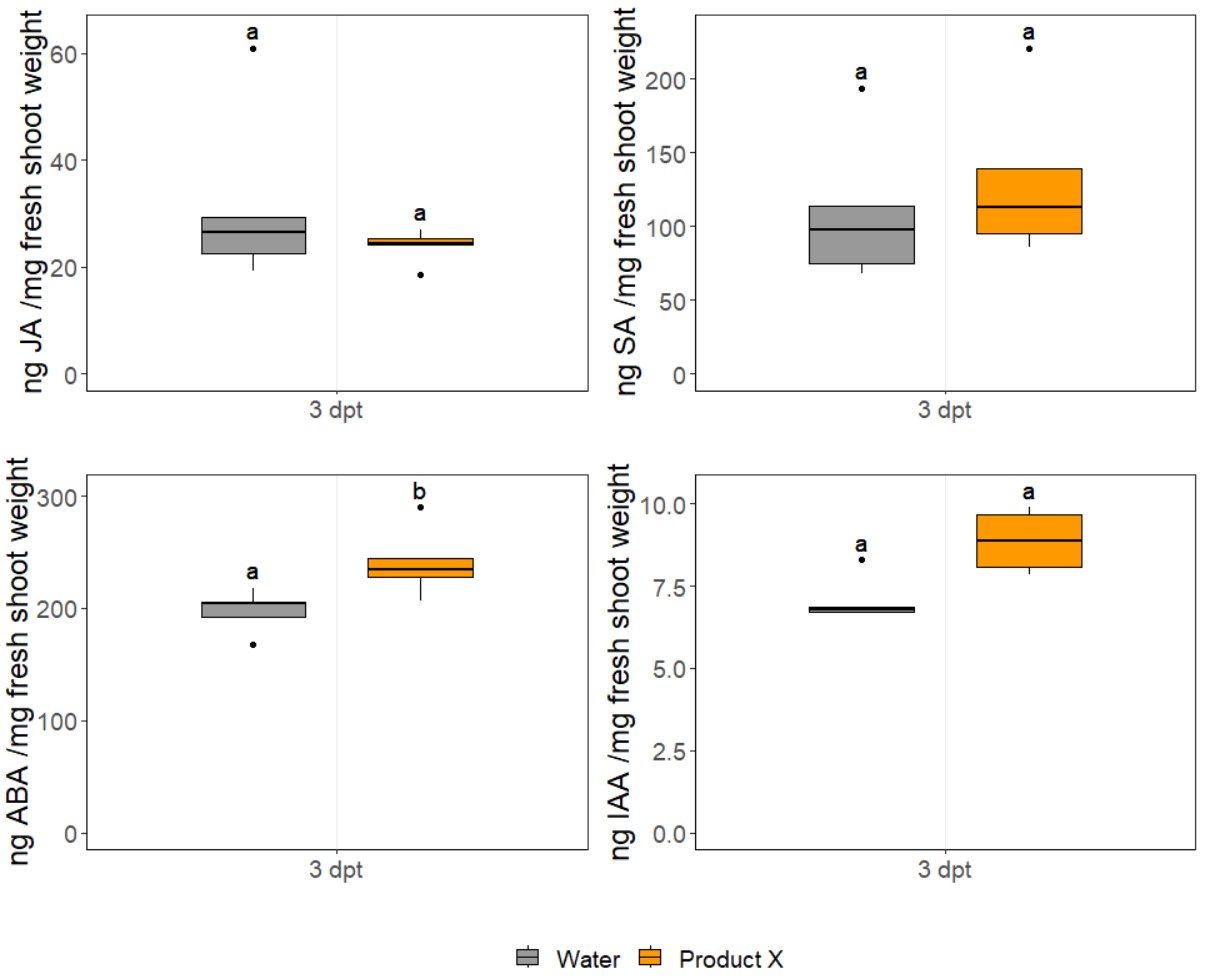
**

*Supplementary Figure 8 – Phytohormone levels at 3 days post treatment (dpt) in shoot material of water and product X treated plants (N=5). Data of control and Product X treated plants was compared per phytohormone.*

# Supplementary Table

*Supplementary Table 1 – GO-analysis of significantly upregulated genes in roots treated with product X. All gene ontology terms are displayed.*

| GO:BP | hydrogen peroxide catabolic process | KEGG | Phenylpropanoid biosynthesis |
| --- | --- | --- | --- |
| GO:BP | hydrogen peroxide metabolic process | GO:BP | response to oxidative stress |
| GO:BP | reactive oxygen species metabolic process | GO:MF | heme binding |
| GO:MF | peroxidase activity | GO:MF | tetrapyrrole binding |
| GO:MF | oxidoreductase activity, acting on peroxide as acceptor | GO:BP | cellular catabolic process |
| GO:MF | antioxidant activity | GO:MF | metal ion binding |
| GO:MF | oxidoreductase activity | GO:MF | cation binding |
| KEGG | Biosynthesis of secondary metabolites | GO:CC | external encapsulating structure |
| GO:BP | catabolic process | GO:BP | detection of hormone stimulus |
| GO:CC | plant-type cell wall | GO:BP | detection of endogenous stimulus |
| KEGG | Metabolic pathways | GO:BP | response to stress |
| GO:MF | small molecule binding | GO:MF | thiamine pyrophosphate binding |
| GO:MF | ion binding | GO:MF | 1-aminocyclopropane-1-carboxylate oxidase activity |
| GO:MF | vitamin binding | GO:MF | catalytic activity |
| GO:MF | oxidoreductase activity, acting on paired donors, with incorporation or reduction of molecular oxygen, reduced ascorbate as one donor, and incorporation of one atom of oxygen | GO:CC | extracellular region |
| GO:CC | cell wall | GO:BP | detection of ethylene stimulus |
